# Highlights from the 2019 International Aspirin Foundation Scientific Conference, Rome, 28 June 2019: benefits and risks of antithrombotic therapy for cardiovascular disease prevention

**DOI:** 10.3332/ecancer.2020.998

**Published:** 2020-01-13

**Authors:** Jaqui Walker, Marco Cattaneo, Lina Badimon, Giancarlo Agnelli, Andrew T Chan, Angel Lanas, Bianca Rocca, Peter Rothwell, Paola Patrignani, Ruth Langley, Gemma Vilahur, Francesco Cosentino

**Affiliations:** 1 International Aspirin Foundation, 34 Bower Mount Road, Maidstone, Kent ME16 8AU, UK; 2 Medicina 2, ASST Santi Paolo e Carlo, Milan, Italy – Dipartimento di Science della Salute, Università degli studi di Milano, 20122 Milan, Italy; 3 Cardiovascular Program-ICCC, IR-Hospital de la Santa Creu, I Sant Pau and CiberCV, 08041 Barcelona, Spain; 4 Internal Vascular and Emergency Medicine-Stroke Unit, University of Perugia, 06123 Perugia, Italy; 5 Clinical and Translational Epidemiology Unit, Division of Gastroenterology, Massachusetts General Hospital and Harvard Medical School; 6 University of Zaragoza, 50009 Zaragoza, Spain; 7 Institute of Pharmacology, Catholic University School of Medicine, Rome, Italy; 8 Centre for the Prevention of Stroke and Dementia and Professor of Clinical Neurology, Oxford, UK; 9 Department of Neuroscience, Imaging and Clinical Sciences, and CeSI-MeT, ‘G.d’Annunizio’ University, School of Medicine, Chieti, Italy; 10 MRC Clinical Trials Unit at UCL, WC1V 6LJ London, UK; 11 Cardiovascular Program ICCC-Research Institute Hospital de la Santa Creu I Sant Pau, IIB-Sant Pau, 08041 Barcelona, Spain and CiberCV, Institute Carlos III, 28903 Madrid, Spain; 12 Unit of Cardiology, Department of Medicine, Karolinska University Hospital, 171 76 Stockholm, Sweden

**Keywords:** aspirin, primary prevention, secondary prevention, colorectal cancer (CRC), cardiovascular disease (CVD), risk, benefit, precision medicine, antithrombotic, antiplatelet, anticoagulant, upper gastrointestinal (GI) bleeding, gastroprotectant agents, optimizing aspirin dose, targeting, diabetes mellitus (DM)

## Abstract

At the 2019 International Aspirin Foundation Scientific Conference ‘Benefits and Risks of Antithrombotic Therapy for Cardiovascular Disease Prevention’, held in Rome, Italy, international experts sought to discuss and debate the optimal antithrombotic strategy for the secondary prevention of cardiovascular disease (CVD) and to seek agreement around dosing and target populations for aspirin use in primary disease prevention. Getting the best evidence to support real-life decisions in the clinic can be complex, and individualising management in order to balance both the risks and benefits of different disease prevention strategies appears to be the best approach. It is hoped that future decision-making tools and biomarkers will help direct treatments at those most likely to benefit.

The International Aspirin Foundation (IAF) 29th scientific conference programme delivered mechanistic data with clinical implications across key areas of debate:

- Antithrombotic therapy in secondary cardiovascular disease (CVD) prevention
  1. Dropping aspirin from dual antithrombotic therapy (DAT) and triple antithrombotic therapy (TAT).
  2. Combining antiplatelet and anticoagulant strategies in high-risk patients.
  3. Reducing upper gastrointestinal (GI) bleeding by more extensive use of gastroprotectant agents.
- Antithrombotic therapy in primary CVD prevention
  1. Optimizing the aspirin dose and dosing regimen
  2. Incorporating other benefits of low-dose aspirin in the benefit/risk equation
  3. Targeting the right population for primary prevention: the case of diabetes mellitus (DM).

**Pippa Hutchison, Executive Director** of the IAF paid tribute to the IAF’s founder, the late Nick Henderson and his work which has enabled scientific debate, research and understanding about aspirin for over four or almost five decades, contributing to many lives saved worldwide.

**Prof. Carlo Patrono, (Catholic University Rome, Italy), Chair of the IAF Scientific Advisory Board** welcomed the audience to Rome and set the tone for a very lively, interactive conference in which multidisciplinary, global experts discussed the wealth of data supporting antithrombotic therapy for CVD prevention.

**Prof. Andrew T Chan (Harvard University, Boston, USA) and Peter Rothwell (University of Oxford, UK)** co-chaired the first session on the benefits and risks of antithrombotic therapy in secondary prevention.

## Dropping aspirin from DAT or TAT

**Prof. Marco Cattaneo (University of Milan, Italy)** presented experimental, mechanistic data to see if there is evidence that DAPT with aspirin and a P2Y_12_ antagonist and monotherapy with a P2Y_12_ antagonist alone had a similar inhibitory effect on platelet function. He showed that whilst pharmacological inhibition of the platelet P2Y_12_ receptors for adenosine diphosphate (ADP) significantly inhibits platelet aggregation and the platelet production of thromboxane A_2_ (TxA_2_) [thromboxaneB_2_ (TxB_2_)], the data also showed that four patients with severe inherited defects of P2Y_12_ had normal serum TxB_2_ levels and one patient with inherited dysfunctional P2Y_12_ also had normal levels of serum TxB_2_.

He explored whether the inhibitory effect of P2Y_12_ antagonists on platelet TxA_2_ production that has been demonstrated in other studies was due to effects such as methodological differences. It is thought that platelet aggregation leads to thromboxane synthesis, which stimulates the release of ADP from the platelet granules. The ADP interacts with its receptors thus amplifying the platelet response and thromboxane production. In order to confirm this hypothesis, platelet aggregation induced by collagen in healthy subjects was tested in stirring conditions. Results showed that in the presence of all P2Y_12_ antagonists, platelet aggregation is reduced compared to the vehicle. TxB_2_ production was measured in the same samples and found to be reduced with all P2Y_12_ antagonists compared to the vehicle. However, when no stirring conditions were used, thromboxane production was not reduced in the presence of the P2Y_12_ antagonists when compared to the vehicle. Therefore, it is the inhibition of platelet aggregation *in vitro* by P2Y_12_ antagonists that is responsible for the observed partial inhibition of TxA_2_ production by platelets.

The effects of the platelet aggregation and level of TxB_2_ after stimulation by high concentrations of collagen in the presence of a P2Y_12_ antagonist (cangrelor) and aspirin, both were explored independently and individually. Results showed the combination therapy of cangrelor and aspirin is better able to inhibit platelet aggregation. The combination therapy produced significant inhibition under experimental conditions.

Prof. Cattaneo explained that P2Y_12_ antagonists, therefore, do not inhibit the platelet production of TxA_2_ and that DAPT with aspirin and a P2Y_12_ antagonist is more effective in inhibiting platelet aggregation than either drug alone, and there is no pharmacological evidence that aspirin is dispensable in high-risk patients who are generally treated with DAPT.

Having established the need for DAPT, Prof. Cattaneo presented further data to help the audience understand how to dose aspirin in DAPT and showed that the effect is best with low-dose aspirin. He concluded that this is probably the lowest dose that causes >95% inhibition of platelet TxA2 production, potentially around 30 mg daily.

**Prof. Marco Valgimigli (University of Bern, Switzerland)** presented the clinical data for removing aspirin from DAT orTAT:

A recent meta-analysis of randomised clinical trials (RCTs) examined the safety and efficacy of DAT compared to TAT in patients with atrial fibrillation (AF) following percutaneous coronary intervention (PCI) [1]. This meta-analysis demonstrated that DAT is better than TAT when looking at bleeding events and comparable for TAT for efficacy outcomes suggesting DAT is the best regimen for the vast majority of patients.

Prof. Valgimigli explored if it was the triple therapy itself or its composition and duration that was the true culprit for the excess bleeding events. He found some issues related to choice of anticoagulant: for instance, warfarin causes more bleeding than a direct oral anticoagulant (DOAC) making this an unfair comparison. In addition, the duration of TAT was often prolonged for longer than the protocol indicated e.g. beyond 6 months which again influenced the results.

The Augustus trial [2, 3] gave important clarification around stroke risk and raised new concerns about stent thrombosis risk where aspirin was dropped from the regimen. The Augustus study showed that dropping aspirin early on was associated with less bleeding but a numerical increase in stent thrombosis events.

In the Augustus study, only patients who actually received a stent were included in the randomisation which is a better measure as in some trials around 50% of patients randomised do not actually receive a stent, which means the risk of a stent getting occluded is non-existent.

‘GLOBAL LEADERS’ [4] is a complex study in which low-dose aspirin (75–100 mg/day) taken for 1 month in patients with either acute coronary syndrome (ACS) or stable angina was followed by 23 months of monotherapy with ticagrelor 90-mg bid. The reference strategy arm received low-dose aspirin for 24 months and the patients with ACS including unstable angina without the cardiac biomarkers, NSTEMI and STEMI received ticagrelor during the first 12 months, whereas the patients with stable angina received clopidogrel 70 mg/day for the first 12 months. This trial had an all-comers design and recruited almost 16,000 patients in a 1:1 randomisation ratio in an open-label design in 130 centres worldwide. GLASSY, a sub set study from GLOBAL LEADERS, showed that 1 month of DAPT was non-inferior to 12 months DAPT when looking at preventing death, myocardial infarction (MI) stroke or urgent target vessel revascularisation.

Data from SMART CHOICE [5] and STOP DAPT-2 [6] were also presented. They showed that dropping aspirin immediately after PCI when used as part of triple therapy cuts the bleeding risk but increases the risk of stent events and dropping aspirin 1–3 months after PCI in the context of dual therapy reduces bleeding risk without a higher ischaemic event risk.

Following the presentations, discussion took place around the types of bleeding events included and how having an MI is often not equivalent to a bleeding event unless this is an intracranial bleed.

## Combining antiplatelet and anticoagulant strategies in high-risk patients

**Figure 1.**
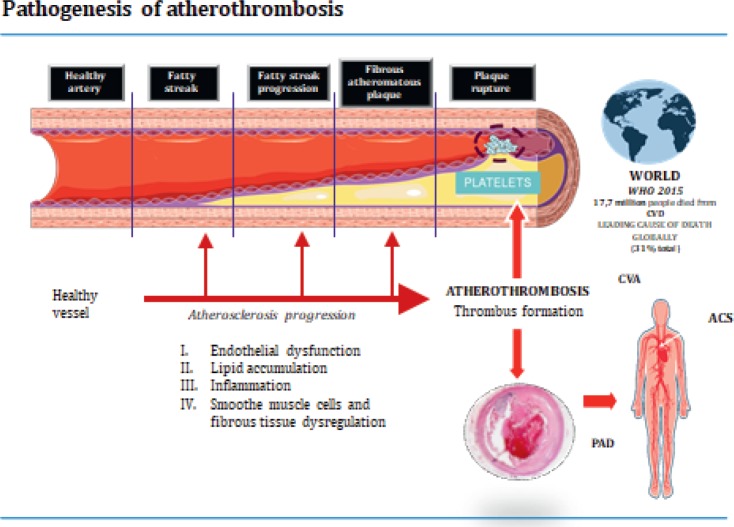
Pathogenesis of atherothrombosis.

**Prof. Lina Badimon (Cardiovascular Research Center, Barcelona, Spain)** explained the science behind the role of platelet activation and blood coagulation in atherothrombosis. Prof. Badimon visually explained the mechanisms of the pathogenesis of thrombosis formation (Figure 1): Platelets released into the bloodstream by bone marrow and circulate for 7–10 days and play an important role in the maintenance of the integrity of vascular walls and in haemostasis. The disruption of an atherosclerotic plaque triggers uncontrolled platelet recruitment, thrombin production and the creation of a thrombus or a clot. In addition, platelets participate in leucocyte and progenitor cell recruitment and may mediate atherosclerosis progression. Extracellular vesicles, such as microvesicles, have a role in all stages of atherosclerotic development and may have a potential use as systemic biomarkers of thrombus growth. It is not just platelets that are involved in the formation of a thrombus; coagulation processes are key to the formation of complex thrombus, as well as white cells and other proteins, such as tissue factor (TF). Prof. Badimon explored the complexities involved in these processes and the importance of understanding what happens within the vessel wall.

Prof. Badimon put this science within the context of patients experiencing a CVD event and explained to the audience the many different parts of the pathway that can be targeted by drug treatment. The delicate balance between CVD risk reduction and bleeding adverse events was also discussed.

It is important to remember that platelets are not only involved in thrombosis; they have other actions in additional systems and/or processes such as angiogenesis, inflammation and cancer, and blocking platelets has important effects in many systems throughout the body.

Despite this detailed insight, Prof. Badimon explained that there is still a lot to learn about how these mechanisms work and interact in order to progress in our understanding of antiplatelet therapy and antithrombotics. It is also important to understand the context in which the clot formation occurs because different reactions take place in relation to different haemodynamic conditions; in a lesion within a high shear vessel, platelets will play a major role but the response is different on lesions in areas of low shear with a higher content of fibrin. Depending upon the haemodynamic conditions, an atherosclerotic lesion may trigger different thrombotic reactions and, therefore, susceptibility to different antithrombotics. The type of atherosclerotic lesion and the haemodynamis conditions are two characteristics behind inter-patient variability in response to treatment.

In the Compass trial [7, 8] inhibition of Factor Xa (an anticoagulant) has a beneficial effect on reducing arterial thrombotic complications. Work is needed to explore other molecules involved including those that block fibrin formation and to increase our understanding on the reasons why one thrombus can fully occlude a vessel while another is only mural.

We must not forget that the coagulation plays an important role in arterial and venous thrombosis.

One particular target of interest has been to block factor II and now factor XI; however, it will be interesting to see if these can show added clinical benefit. Understanding more about the different types of thrombi may have an impact on clinical management in the future.

The differences in plaque rupture and plaque erosion in the clinical setting were discussed. The evidence indicates a sex difference with more women experiencing plaque erosion and men having more ruptures in relation to age. As women get older, however, more plaque rupture can occur. It seems smokers and younger patients have more plaque erosions. Our knowledge of women’s cardiovascular health, an area of unmet clinical need, is expanding and the European Society of Cardiology (ESC) is currently aiming to increase our understanding of CVD in women promoting their participation in the new large clinical trials.

Prof. Badimon concluded that the future will bring a more targeted approach to CVD management and noted that two important areas for development are: (a) a greater understanding of the variable thrombotic response to atherosclerotic plaque disruption and (b) the decipher the delicate balance of safety and efficacy by promoting the development of drugs without haemorrhagic risks.

**Prof. Giancarlo Agnelli (University of Perugia, Italy)** complimented Prof. Badimon’s talk with an analysis of the trial data in which antiplatelet regimens with low-dose aspirin plus or minus a thienopyridine (selective, irreversible ADP receptor/P2Y_12_ inhibitor) is combined with the anticoagulant low-dose rivaroxaban. Two clinical settings for this approach were explored:

- recent ACS (1–7 days after a hospital admission for ACS)
- stable atherosclerotic vascular disease; coronary artery disease (CAD) and peripheral artery disease (PAD).

ATLAS ACS TIMI 46 [9] is a randomised, double-blind, placebo-controlled, dose-escalating study of 3,491 patients with a primary outcome of clinically significant bleeding. Different doses of rivaroxaban, 5, 10, 15 and 20 mg (with a twice-daily dose when doses less than 10 mg were used to ensure 24-hour coverage), were used in combination with aspirin alone or aspirin plus clopidogrel. The study found that while the addition of rivaroxaban reduced the risk of death, MI or stroke, there was an increased risk of bleeding events that required medical intervention and in order to reduce the risk of bleeding a lower dose of rivaroxaban is required and the twice-daily dose appeared to be slightly safer.

ATLAS ACS 2 TIMI 51 [10], a double-blind, event-driven study in which aspirin at a dose of 75–100 mg per day, with or without thienopyridine plus either placebo, rivaroxaban 2.5 mg twice daily or rivaroxaban 5 mg twice daily was studied in 15,526 patients. The primary efficacy end point was a composite of death from either a CVD cause or stroke. This study found that the addition of rivaroxaban did reduce the risk of death from the composite end point of cardiovascular causes, MI or stroke, and that although rivaroxaban did increase the risk of major bleeding and intracranial haemorrhage, its addition to the antithrombotic regimen did not increase the risk of fatal bleeding. It was the lower dose of 2.5 mg of rivaroxaban taken twice daily that showed a survival benefit with a number needed to treat 49.

These two studies clearly showed that very low doses of rivaroxaban, an oral anticoagulant, are a useful addition to anti-platelet, antithrombotic regimens aimed at reducing subsequent CVD events in people with ACS.

**Figure 2.**
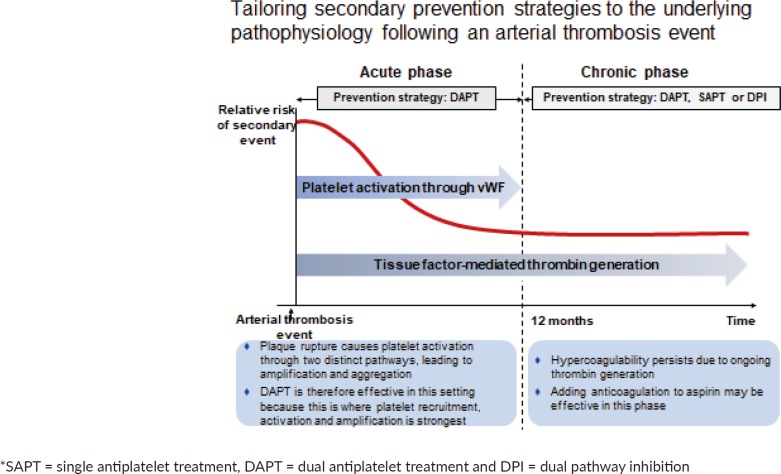
Tailoring secondary prevention strategies to the underlying pathophysiology following an arterial thrombosis event.

In stable, atherosclerotic vascular disease, there is a substantial overlap between patients with CAD and PAD [11], and this increased polyvascular disease is associated with an increased risk of morbidity and mortality. A residual risk of vascular events despite optimal anti platelet therapy is present in patients with chronic CAD or PAD [12–16]. Secondary prevention could be tailored to an understanding of the underlying pathophysiology after an arterial thrombus event (Figure 2).

The COMPASS study aimed to explore how patients with multivessel disease are more at risk of CVD morbidity and mortality can be better protected from a secondary event with antithrombotic therapy [17]. COMPASS showed that in this patient population with stable atherosclerotic vascular disease, those given rivaroxaban (2.5 mg twice daily) plus aspirin did have better cardiovascular outcomes [24% lower CVD death, stroke or MI (4.1% rivaroxaban 2.5 mg twice daily plus aspirin versus 5.4% rate just aspirin)] than those given aspirin alone but that this was at the cost of a higher rate of bleeding events [17].

Prof. Agnelli explained that, while there is a benefit in terms of reduced CVD events when adding rivaroxaban to aspirin for secondary prevention, it is important to use clinical judgement to select the right patients. The combination of antiplatelet anticoagulant approach is more expensive and needs careful prioritisation to those who are most likely to benefit. In practice, Prof. Agnelli suggests those will be who are at high risk of a cardiovascular event who do not have an increased risk of having a bleed, e.g. a person with multi-vessel involvement but no risk factors for bleeding with antithrombotic therapy.

## Reducing upper GI bleeding by more extensive use of gastro protectant agents

**Prof. Andrew T Chan (Harvard University, Boston, USA)** delivered a comprehensive and clear insight into the mechanism behind the upper GI complications that are induced by antithrombotic drugs**.**

Prof. Chan described epidemiological factors influencing aspirin and adverse GI bleeding events. Most of the bleeding occurs within the first 6 months of regular aspirin use [18] and is strongly associated with higher dose rather than longer duration of treatment [19, 20]. He also explained that aspirin may be associated with a higher relative bleeding risk when used for primary rather than secondary CVD prevention but this is balanced by the lower baseline risk within those taking aspirin for primary prevention [21].

As we grow older, our risk of a serious bleed increases with regular aspirin [22]. *Helicobacter pylori* (*H. pylori*) infection also increases the risk of having a gastroduodenal ulcer and gastric bleeding in those taking regular aspirin [23]. It was also noted that *H. pylori* eradication can increase compliance with low-dose aspirin as gastric intolerance such as dyspepsia and reflux is reduced. Later in the conference, the prevalence rate of *H. pylori* was discussed; this varies from approximately 10%–20% in UK, USA and Sweden to around 70% in some Middle Eastern countries. In the case of primary prevention, there is time to screen for *H. Pylori* before treatment commences, whereas in secondary prevention the *H. pylori* test may need to carried out after the patients has started treatment. If testing is positive *H. pylori* can be eradicated and thus help to reduce the risk of GI side effects. Other risk factors for upper GI complications with regular aspirin or other non-steroidal anti-inflammatory drugs (NSAID) include the following.

- Previous peptic ulcer or GI bleed
- Concurrent use of two or more NSAIDs
- Concurrent use of a corticosteroid
- Concurrent use of an anticoagulant
- Presence of severe disease [24].

It is hoped that tools to estimate the individualised risk of CVD events to be avoided versus bleeding events caused by aspirin initiation will be developed. Work has been carried out in New Zealand on the 385,191 individuals who received a CVD risk assessment between 2007 and 2016 and has shown a predicted 5-year bleeding risk of 1% in women and 1.1% in men [25].

**Figure 3.**
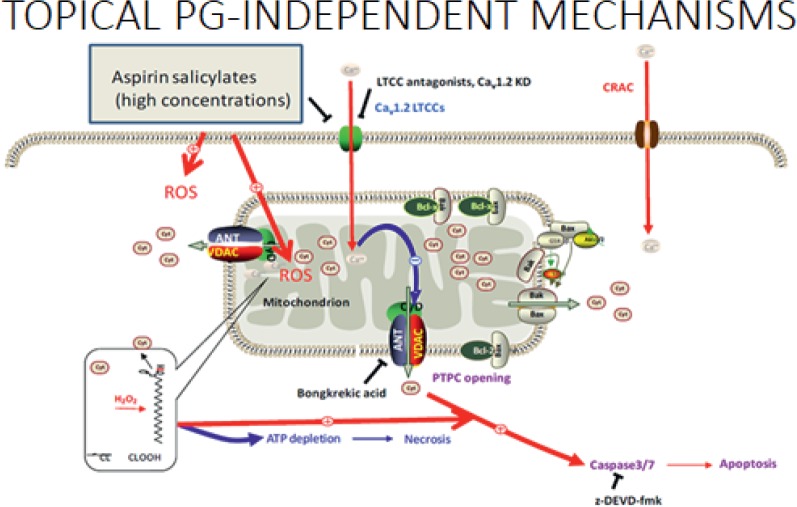
Topical PG-independent mechanisms.

Next, Prof. Chan explained how aspirin causes mucosal injury by inhibiting the usual gastro protective mechanisms of prostaglandins (PGs) and has additional PG-independent local actions which result in direct injury and death of epithelial cells. This occurs as a result of aspirin changing the acid balance in the gastric mucosa (mostly in the small and large bowel) damaging tight-junctions and exposing the gastric mucosa to the gut content [26, 27]. Other ways in which aspirin has a topical effect on gastric mucosa include its ability to penetrate cells and decrease the epithelial surface hydrophobicity making the cells more susceptible to injury [28]; the deacetylation of aspirin to salicylate is cytotoxic [29] and aspirin can alter the local microcirculation resulting in tissue injury [30]. In this way, the topical PG-independent mechanisms of aspirin have effects throughout the GI system (Figure 3). It has only a minor effect on the stomach/duodenum and enteric-coated aspirin does not significantly alter the risk of a gastroduodenal ulcer. The more significant topical effect of aspirin on GI tract bleeding takes place in the distal small bowel and large bowel [31, 32].

However, it is the systemic effects of aspirin that are most important for its GI side effects and these side effects are not reduced when presentations such as enteric-coated aspirin [32], or different routes of administration of NSAIDS, e.g. IM or IV are used [33, 34].

Aspirin works systemically to inhibit PG synthesis and platelet activation which is why it can also cause bleeding adverse events. Aspirin and NSAIDS as a group block the cyclo-oxygenase (COX) enzymes that are responsible for PG synthesis thus impacting on the body’s gastro protective mechanisms. Platelet activation is also vital for clot formation and the production of growth factors that help tissue to heal.

Other non-aspirin, anti-platelet drugs such as the P2Y_12_ inhibitor agents or thienopyridines (clopidogrel, ticagrelor and prasugrel) also cause GI bleeding due to reduced growth factor production and increased risk of bleeding due to an impaired clotting mechanism [34]. Some people with a genetic variation are carriers of the CYP2C19*17 allele and have excessive platelet inhibition when taking thienopyridines. These individuals are more likely to develop bleeding complications with thienopyridines therapy [35, 36]. Other factors that influence the bleeding risk with P2Y_12_ receptor antagonists include: diabetes, age, smoking, weight and drug interactions [37, 38]. In contrast to aspirin P2Y_12_ receptor, antagonists are probably not the main cause of peptic ulcer by themselves but they potentiate them by their anti-platelet activity and altered angiogenesis [34].

The protease-activated receptor–1 (PAR-1) antagonist, vorapaxar which is effective for the secondary prevention of CVD in patients with impaired renal function when added to aspirin or another anti-platelet agent significantly increases the risk of major bleeding [39, 40].

Therefore, aspirin increases the risk of GI bleeding by both topical and systemic mechanisms and the newer anti-platelet agents carry a similar or slightly higher bleeding risk. Combination antiplatelet regimens increase the risk of having a bleed further with aspirin plus clopidogrel having a higher bleeding risk than aspirin alone and triple/quadruple regimens further exacerbating the overall bleeding risk. Adding a proton pump inhibitor (PPI) and/or a histamine 2 receptor antagonist (H_2_RA) can reduce the risk of upper GI bleeding [32, 22].

Concerns that PPIs may block some of the antithrombotic effect of clopidogrel appear to not be clinically important but are still of enough concern for the FDA to warn people to consult their clinician before staring a PPI if they are prescribed clopidogrel. The number needed to treat with a PPI in order to avoid a disabling bleed is over 300 in younger populations but this figure drops to around 25 in older adults. It is important to carefully consider the likely risk versus benefit of each antiplatelet and how many antiplatelets are required when selecting a treatment regimen for patients [41]. Platelet function tests may help to facilitate the choice of antiplatelet therapy [42, 43]. De-escalation of combination antiplatelet regimens will be also considered important [44].

**Prof. Angel Lanas (University of Zaragoza, Spain)** in a complementary clinical talk presented data from clinical trials of gastroprotectant agents used with antithrombotic therapy. Despite being the cornerstone for the prevention of CVD, antiplatelet and anticoagulant drugs are associated with a two- and fourfold increased risk of GI bleeding, respectively. The newer DOACs also increase the risk of gastric bleeding [45] the risk increases when antithrombotic therapies are combined and when the patient has other risk factors for a bleeding event. Low-dose aspirin (75–100 mg daily) is the most widely prescribed antiplatelet drug and endoscopic trials have shown that PPIs are effective in treating and preventing gastroduodenal ulcers associated with its use. The H_2_RA famotidine is also effective at reducing gastric bleeds with aspirin but there is less consistent data to support its use, and studies indicate that PPIs have superior efficacy [46].

**Figure 4.**
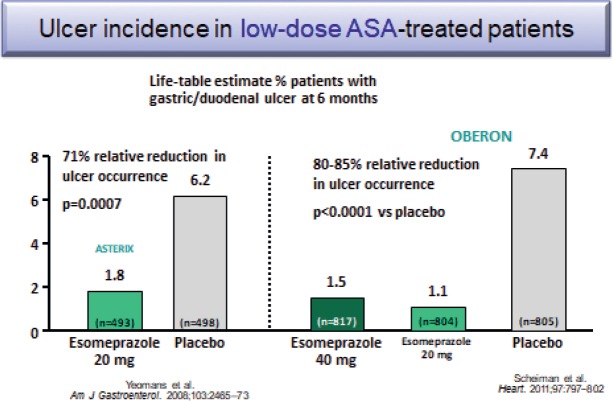
Ulcer incidence in low-dose ASA-treated patients.

Several studies have shown that PPI co-therapy with either 20 or 40 mg of esomeprazole reduce by 71%–85% the occurrence of peptic ulcers associated with low-dose aspirin use (Figure 4) [47, 48].

**Figure 5.**
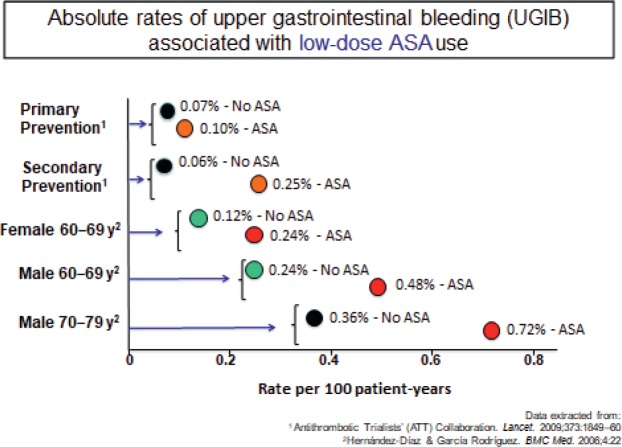
Absolute rates of upper gastrointestinal bleeding (UGIB) associated with low-dose ASA use.

To put the risk into context, Prof. Lanas explained that based on a meta-analysis of RCTs published by the Antithrombotic Trialists’ (ATT) collaboration [12] and on estimations based on epidemiological studies, in primary prevention, aspirin use was associated with three extra GI bleeds for each 10,000 patient-years and 19 extra cases per 10,000 patient-years in secondary prevention (Figure 5).

**Figure 6.**
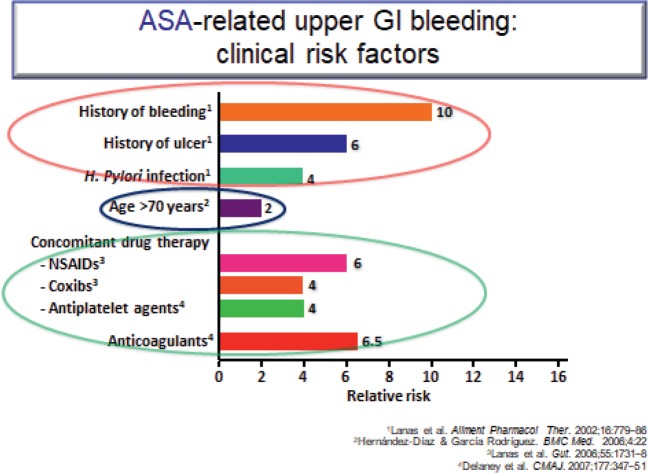
ASA-related upper GI bleeding: clinical risk factors.

The ATT [12] meta-analysis of RCTs interestingly showed that it is the well-known CVD risk factors such as age, male gender, diabetes, smoking, increased blood pressure (BP) or obesity that are also risk factors for GI bleeding (Figure 6). In high-risk patients with a previous ulcer, PPIs are effective in reducing a recurrence. Omeprazole (PPI) has been shown to be effective in preventing upper GI complications for patients on dual anti-platelet therapy. The benefit of using antisecretory drugs such as a PPI or H_2_RA to prevent GI bleeding when anticoagulants are used alone or in combination with antiplatelets is as yet unproven.

However, antisecretory agents have no effect on GI damage prevention associated with antithrombotic agents in the small bowel. Further investigation is needed with ‘mucosal protectant’ or other agents. Other future areas of research interest include the gut microbiome and studies already under way to increase understanding around how the gut microbiome affects bleeding risk, and if there is a role for treatment here such as probiotics. This will be, especially, useful if ways to protect the small bowel from bleeding risk can be developed.

Misoprostol exhibits moderate efficacy in healing small bowel ulcers associated with a previous bleeding event. Further research is needed.

Prof. Lanas concluded that preventing gastric bleeding with antithrombotic regimes is an important area of unmet clinical need and will grow in importance with the current increasingly elderly population needing these agents. PPIs have been shown to be effective at preventing peptic ulcers and peptic ulcer bleeding in patients taking low-dose aspirin. PPIs are better than famotidine (H_2_RA) in this effect.

**Prof. Michael Gaziano (Harvard University, Boston, USA)** and **Prof. John Chia (NCCS, Singapore)** chaired the afternoon session in which the benefits and risks of antithrombotic therapy in primary prevention were debated.

## Optimising the aspirin dose and dosing regimen

**Prof. Bianca Rocca (Catholic University, Rome, Italy)** explained inter-individual variability in the extent and duration of platelet thromboxane inhibition by low-dose aspirin. Understanding interindividual variability of drug responsiveness is important for improving drug effectiveness and safety in the clinical setting and the key objective for precision medicine. Finding ways to better understand what determines variation in aspirin response is important for finding new ways to administer an ‘old’ yet effective, cheap drug in the optimal way.

**Figure 7.**
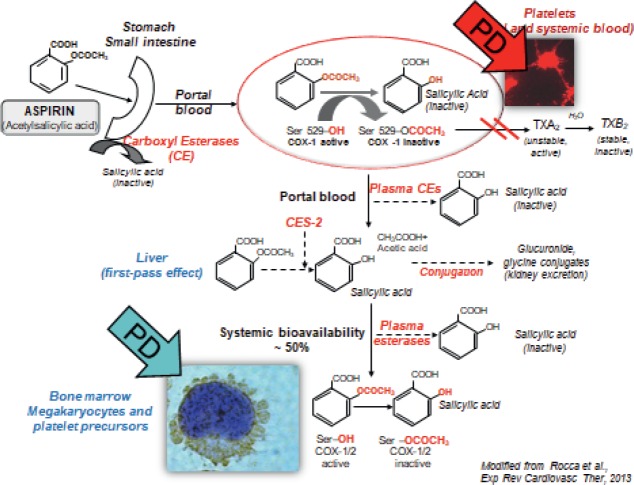
The pharmacokinetics and pharmacodynamics of aspirin in the human body.

In a world where patients increasingly have co-morbidities and polypharmacy the fact that aspirin is not metabolised via the CYP450 system is advantageous as this means there is little impact from drug–drug interactions at a pharmacokinetic level on drug responsiveness. Patient characteristics, however, including other diseases can alter the responsiveness to aspirin. In its pre-systemic availability aspirin, before it passes the liver, inhibits circulating platelets by irreversibly blocking the COX-1 enzyme. Based on systemic bioavailability, aspirin acts to inhibit the precursors of platelets in the bone marrow such as megakaryocytes, pro and pre platelets (Figure 7).

Chronic conditions including diabetes, obesity or myeloproliferative neoplasms can influence the extent and/or duration of platelet COX-1 inhibition and TXA_2_ generation through disease-specific pharmacokinetic or pharmacodynamics mechanisms. A twice daily low-dose dose regimen restores adequate platelet COX-1 inhibition without effecting vasculo-protective endothelial prostacyclin in conditions with chronically enhanced platelet generation. Serum TXB_2_ *ex vivo* can act as a pharmacodynamic biomarker surrogate of aspirin efficacy and is used for the approval of new aspirin formulations.

It is hoped that an in silico model of low-dose aspirin pharmacokinetics and pharmacodynamics will help the design of personalised regimens, the so-called ‘precision dosing’, that can then be tested in adequately sized clinical trials of improved versus conventional antiplatelet regimens. This will help build information around how to dose aspirin in patients with moderate-to-severe obesity and/or rare diseases such as essential thrombocythaemia (ET), which are infrequent in or even excluded from traditional randomised controlled trials. It will also help guide aspirin dosing in complex clinical scenarios such as patients with hepatic or kidney impairment, pregnant women, elderly and frail subjects, with different CVDs. The model has an ability to learn when challenged with different real-world data and can be built so that it will be able to advice on aspirin dosing in different individuals, combining different clinical conditions and antropometric features in the future.

Myeloproliferative neoplasms such as ET (where changes within the megakaryocytes lead to an increase in thrombotic events) and Polycythemia Vera (a blood cancer in which the bone marrow makes too many red blood cells causing the blood to become too thick) are associated with a 3.5-fold increase in serious vascular events even with standard low-dose aspirin [49]. Prof. Rocca explained how newly released platelets with unacetylated COX-isozymes released over the traditional dosing interval of once in 24 hours may contribute to the variable lower aspirin responsiveness in ET and that more frequent dosing may be needed. The aspirin regimens in essential thrombocythaemia: (ARES) phase II trial has been set up to test more frequent dosing strategies for aspirin in ET. Initially, twice daily and three times a day regimens will be explored with the best regimen taken forward to the second part of the study, when serum TXB_2_ measures will be taken every 3 months in order to assess the long-term persistence of superior biochemical efficacy and safety of the optimised dosing regimen.

Currently, we rely on consensus expert advice to guide these clinical scenarios, for example, the ESC Working Group on Thrombosis consensus statement on aspirin and obesity, which advices that while limited data is available on aspirin dosing in people with a body mass index (BMI) above 40 kg/m^2^, it is reasonable to double the daily low dose of aspirin or give a twice-daily low dose. Recent work has shown a higher residual TXB_2_ in moderate-to-severe obese people in contrast to non-obese subjects in their response to once-daily low dose aspirin, and in fact, increasing residual serum TXB_2_ was found to be exponentially correlated with BMI and body weight in 100 subjects taking standard low-dose aspirin (100 mg once daily) [50].

Prof. Rocca concluded that this is just the beginning of the story and that a lot more research and development will be required. Aspirin resistance does not exist; it is low compliance and inter-individual sources of variability that affect the extent of drug response. At variance with drug resistance, which implies the loss of drug effectiveness even by increasing dose, variability can be corrected in the context of precision dosing and medicine

**Prof. Peter Rothwell (Centre for the Prevention of Stroke and Dementia, Oxford, UK)** then looked at the clinical trial data for different aspirin doses, dosing regimens and compliance with treatment in order to explain the effects this can have on the effectiveness of treatment in clinical practice. In particular, he sought to answer how we can estimate the likely effect of aspirin in clinical practice for patients who take the drug regularly.

Clinical trials of aspirin in the prevention of vascular events are quite heterogeneous and trying to come up with a simple answer to a complicated question from pooled analyses can be over-simplistic. Trials investigating aspirin’s effect on the prevention of vascular events have also evolved over time. Secondary prevention trials in the 1980s used high-dose aspirin in a mostly male smoking or ex-smoker population; early primary prevention trials were also done in men only, usually at age less than 70 years and mostly with low BMI; more recent primary prevention trials included women there were fewer smokers and a more mixed BMI profiles and the most recent primary prevention trials from 2010 onwards have tended to look at special populations with generally higher BMI such as diabetics (ASCEND), patients with a high vascular risk (ARRIVE) and older patients (ASPREE). Additionally, the wider use of statins and BP-lowering drugs has also changed the primary care prevention landscape.

The clinical trial setting is also different from clinical practice. The intention-to-treat analysis is important for limiting bias, but results can mislead due to compliance issues. This makes on-treatment analysis of some interest but this raises issues of bias and loss of randomised groups. In contrast, time-course analysis can be helpful in understanding the effects of increasing treatment drop-out and drop-in overtime during follow-up whilst still retaining the intention to treat analysis and randomised groups. Prof. Rothwell reviewed the available data on time-course analysis of the effects of aspirin in randomised trials. The data suggest that patients who take aspirin for primary prevention of CVD need to have good compliance rates in order to reap benefits over time.

Ongoing trials of different aspirin doses or dosing regimens were also reviewed. ADAPTABLE is a pragmatic trial of patients at high risk of ischaemic events. Patients are randomised to receive either 81 or 325 mg of aspirin daily in a non-blinded open-label manner. The study aims to recruit 15,000 participants with known atherosclerotic CVD enrolled over 36 months with a maximum follow up of 40 months. The primary outcomes are all-cause death and hospitalisation for MI or stroke. The primary safety endpoint is hospitalisation for a major bleeding event with a blood transfusion. The study commenced in April 2016 and aims to complete it by June 2020.

Another new study, ANDAMAN, has been designed to look at aspirin twice daily in patients with diabetes and ACS. The study will compare a dose of 100 mg enteric-coated aspirin given in the evening with a twice-daily 100 mg AM and PM dose. The primary outcome for this study is the first serious vascular event occurring within 18 months after randomisation. The study which started in 2016 is due to reach its final data collection for the primary outcome measure in February 2020.

These trials along with updated data to pre-existing analysis with recent trial data should help to produce the evidence base that dose and dosing regimen is important in terms of clinical outcomes for the antithrombotic effects of aspirin. It appears that ‘one dose fits all’ approach may not be appropriate for aspirin in the prevention of vascular events or cancer and factors such as body weight and/or BMI may need to be taken into account.

## Incorporating other benefits of low-dose aspirin in the benefit/risk equation

**Prof. Paola Patrignani (‘G. d’Annunzio’ University of Chieti, Italy)** explained the mechanisms underlying the non-vascular effects of low-dose aspirin**.** The data for a chemopreventive role for low-dose aspirin against colorectal cancer (CRC) and other cancers is growing. This effect appears to be from the prevention of early neoplastic transformation within the gut and an anti-metastatic action. The antiplatelet activity of low-dose aspirin can mostly explain both of these effects. Aspirin acts by acetylating platelet COX-1 at a critical serine residue (Ser-529) close to the catalytic site of this enzyme.

As a result of *in vitro* studies, using supratherapeutic concentrations, various mechanisms for aspirin anti-cancer action have been proposed:

- Inhibition of nuclear factor kappa B (NF-kB) signalling
- Interruption of extracellular signal-regulated kinases (ERK)
- Inhibition of Wnt/b-catenin signalling
- Capacity of aspirin to acetylate extra-COX proteins, e.g. p53
- Activation of adenosine monophosphate-activated protein kinase.

Low-dose aspirin can also partially acetylate rectal mucosal COX-1, associated with the reduction in PG E2 and phosphorylated S6 (p-S6) levels. This effect might interfere with early colorectal carcinogenesis [51, 52].

Platelets sustain cancer cell invasion and metastasis formation by supporting the development of epithelial–mesenchymal transition (EMT), cancer cell survival in the bloodstream and the enhancement of tumour cell adhesion to the endothelium facilitating arrest and extravasation. Platelets also contribute to tumour escape from immune elimination.

Aspirin, by inhibiting platelet activation triggered by GI mucosal lesions, restrains the development of chronic inflammation, which is crucial in cancer development. Aspirin can also acetylate COX-1 expressed in colorectal mucosa leading to changes in the mucosal phenotype.

The interaction of cancer cells with platelets results in the following molecular and functional consequences:

- Induction of COX-2 in CRC cells (a hallmark of cancer)
- Induction of EMT in CRC cells promoting migration and metastatic potential
- Induction of a prothrombotic phenotype in cancer cells
- Enhanced systemic biosynthesis of TXA2 [53, 54].

**Figure 8.**
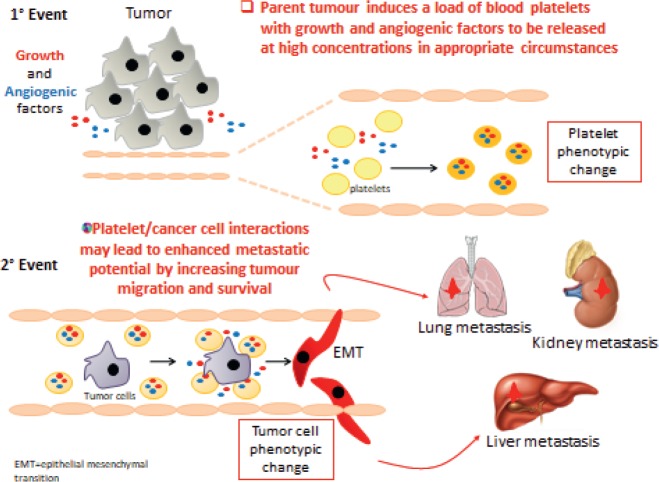
The role of platelets in cancer cell invasion and metastasis formation.

Evidence is now accumulating to support the hypothesis that activated platelets contribute to CRC development and metastatic spread by direct cell to cell interactions with stromal cells and cancer cells, together with the release of different lipid and protein mediators and microvesicles (Figure 8). Platelets can also uptake proteins and genetic material that is present within the bloodstream. Evaluating the proteomics and transcriptomics signature of platelets and platelet-derived microparticles may present a new strategy for developing biomarkers for early cancer detection and for therapeutic drug monitoring in cancer chemotherapy.

**Prof. Ruth Langley (University College London, UK)** presented an overview of the clinical trial evidence for aspirin’s nonvascular, cancer chemopreventive effect.

The talk was divided into two areas:

- Aspirin for the prevention of primary cancers and progression of pre-malignant lesions
- Aspirin for the prevention of metastases and recurrence after radical therapy.

The evidence for aspirin’s nonvascular effects comes from pre-clinical work, epidemiological studies and randomised trials including those that were initially designed to assess the vascular effects of aspirin [55–57].

In terms of primary prevention, recent studies include SeaFOod where mean colorectal adenomas per patient were reduced by aspirin particularly right-sided, serrated lesions supporting previous data [58] and the AspECT trial which showed that high dose PPIs and aspirin compared to low dose PPI/no aspirin for patients with Barrett’s oesophagus increased time to event defined as high-grade dysplasia, oesophageal adenocarcinoma and all-cause mortality [59]. The CAPP2 trial [60] has already demonstrated that patients with Lynch syndrome benefit from aspirin and results are awaited from the CAPP3 trial which recently completed recruitment and is focussed on the dose of aspirin required for anti-cancer effects.

Results from the ASPREE trial [61] (aspirin in an elderly population) were unexpected. The primary outcome measure was a composite of death, dementia and permanent physical disability, with a follow up of 4.5 years 21.5 versus 21.2 events per 1,000 person-years were seen with aspirin compared to placebo (HR 1.01, 95% CI 0.92–1.11). However, an increase in mortality with aspirin was noted which has been attributed to an increase in the risk of metastatic cancer but not incident cancers overall. This might be accounted for by aspirin unmasking malignant disease through an increased risk of bleeding particularly as the incidence and severity of bleeding with aspirin increase with age.

A number of clinical trials are also underway to evaluate the role of aspirin as an adjunct therapy for colorectal, breast and other cancers (ASCOLT, ASPIRIN, US Aspirin Breast Cancer (ABC) trial, PIK3CA based trials and the Add-Aspirin trial). The PIK3CA mutation trials are based on epidemiological data that aspirin after a diagnosis of CRC is most effective if the cancer has a PIK3CA mutation [62]. The Add-Aspirin trial is recruiting patients from the UK and India and aims to assess the addition of either 100 or 300 mg of aspirin after standard primary therapy for early-stage breast, colorectal, gastro-oesophageal and prostate tumours. Over 7,000 patients have been recruited to this trial and will be followed for at least 5 years with long-term passive follow-up via the National Cancer Intelligence Network (NCIN) in the UK [63]. Adherence in this study has been good with 95% of participants taking six to seven of the tablets per week. Data from the run-in period of the trial (aspirin 100 mg open-label for 8 weeks) from over 2,000 patients has shown a low toxicity rate with only 13/2,253 (0.6%) of the participants having had a grade 3+ toxicity.

Prof. Langley concluded by stressing the importance of long-term follow-up in both primary and adjuvant aspirin cancer trials and noted the importance of electronic health records for this work. She also commented that the benefit/risk ratio for aspirin in terms of cancer prevention is likely to favour aspirin use in a ‘younger’ (middle-aged rather than elderly) population. Cancer Research UK (CRUK) recently funded an international collaboration to further explore the role of aspirin in cancer prevention with the aim of understanding the mechanism of action. Finally, the concept of cancer chemoprevention in primary, secondary and tertiary approaches needs wider promotion and greater public awareness.

More details of the lecture and trials are available on the Aspirin Foundation website (https://www.aspirin-foundation.com/conferences-2/scientific-conference-2019-2/).

## Targeting the right patient population for primary prevention: the case of diabetes mellitus

**Figure 9.**
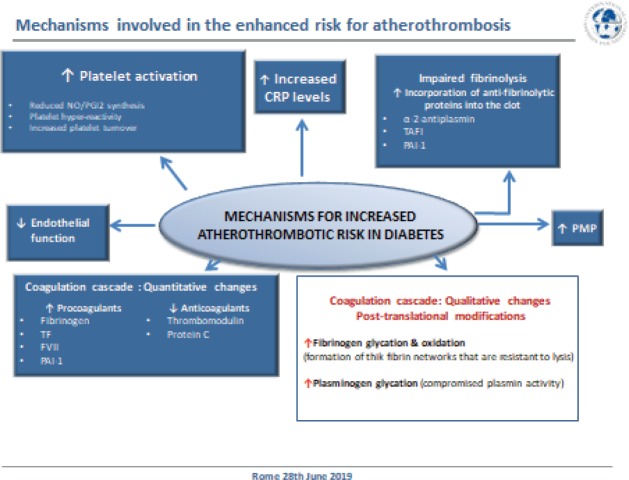
Mechanisms for increased atherothrombotic risk in diabetes.

In the final paired session of the day, **Dr. Gemma Vilahur (Cardiovascular Program ICCC – Research Institute Hospital Santa Creu i Sant Pau, Barcelona, Spain)** explored the mechanisms of atherothrombosis in DM (Figure 9). DM is a global emergency and represents a major cause of cardiovascular morbidity and mortality in developed countries with atherothrombosis being the most common cause of death. It is estimated that 8/10 individuals with DM will die from a cardiovascular event. Multiple pathophysiological mechanisms are thought to account for the pro-atherosclerotic and pro-thrombotic status associated with diabetes, such as obesity, dyslipidemia, and low-grade inflammation. Diabetic patients display a less thromboresistant vasculature mainly because of impaired endothelial function and a consequent decline in the production of key protective molecules, including nitric oxide (NO) and prostacyclin. In addition, the platelets of diabetic patients have an enhanced turnover, they have over-production of thromboxane and exhibit a hyper-reactivity phenotype derived from a dysregulation at both the receptor and the intracellular signal transduction levels. On top of this, quantitative and qualitative changes of haemostatic factors account for the diabetic hypercoagulable state which, in concurrence with impaired endogenous fibrinolysis, leads to denser clot structures and delayed spontaneous clot lysis.

Preliminary studies using intracoronary angioscopy have demonstrated that patients with T2DM not only had an increased incidence of ruptured atherosclerotic plaques in their coronary circulation, but there was virtually a doubling of intravascular thrombi in these patients, suggesting an abnormal tendency towards thrombus formation or clot dissolution [64].

The endothelium, a single layer of cells in the interior of blood vessels, provides a physical barrier. Its main purpose is to maintain vascular homeostasis regulate vascular tone and smooth muscle cell proliferation, reduce inflammation and prevent thrombosis [65]. Endothelial dysfunction can thus be used as a critical surrogate endpoint for CVD [66–68]. Insulin resistance and sustained hyperglycemic levels experienced with DM directly affect endothelial activity, inhibiting insulin-mediated protective mechanisms, and stimulating maladaptive responses. In its normal protective and physiological state, insulin interacts with insulin receptors leading to the synthesis of two important vasodilators and antithrombotic agents, NO and prostacyclin (PGI2) [69–71]. Insulin resistance with DM diminishes these key protective mechanisms resulting in reduced vasodilation and the loss of antiplatelet effects. In addition, hyperglycaemia leads to an increase in systemic oxidative stress (reactive oxygen species (ROS) production) [72]. This enhanced ROS production compromises NO synthesis and stimulates endothelial inflammation via several cellular mechanisms, including promoting activation of PKC and NF-κB signalling. NF-κB, in turn, induces the transcription of inflammatory response-associated genes [73] and vascular adhesion molecules further stimulating leukocyte recruitment, thereby aggravating the inflammatory process [74]. ROS also reduces NO levels by eNOS downregulation and induces the formation of highly oxidant peroxynitrite ion and asymmetric dimethylarginine (ADMA), an endogenous competitive inhibitor of eNOS activity. Diabetic patients not only produce an excess of ROS but their antioxidant mechanisms are also impaired (e.g. reduced superoxide dismutase) [73, 75]. The prolonged oxidative response that occurs during hyperglycaemic states results in the formation of advanced glycation end products (AGEs) by glycosidation of proteins and fatty acids [74]. AGE interaction with its receptors (RAGE) maintains endothelial dysfunction by promoting the release of pro-inflammatory cytokines and cell adhesion molecules, compromising the endothelial barrier function and leading to increased vascular leukocyte infiltration. Finally, recent data have suggested that red blood cells also contribute to DM-related endothelial dysfunction by increasing endothelial arginase-1 activity, an eNOS competitor for L-arginine substrate [76, 77].

Multiple factors are involved in the increased platelet reactivity observed in DM patients [78]. Hyperglycaemia increases Ca^2+^ mobilisation from intraplatelet storage pools leading to an increased intracellular Ca^2+ level^ and the consequently enhanced sensitivity to aggregating agents. People with DM have impaired insulin receptors [71] and the altered signalling of these platelet insulin receptors promotes the expression of adhesion molecules (GpIIb/IIIa, P-selectin) and prothrombotic agonists (thrombin, ADP, TxA_2_). Platelets from people with DM synthesise more TxA_2_ than normal platelets in response to a variety of agonists that induce deacylation of arachidonate from membrane phospholipids and show hyper-responsiveness of proteinase-activated receptor (PAR) to thrombin and of the P2Y12 receptor to ADP [71, 78]. In addition, DM patients have an enhanced platelet turnover and this increases the proportion of highly reactive, newly formed platelets with lower platelet fluidity due to changes in membrane lipid structure or glycation of membrane proteins and with alteration in its intracellular components [79].

Dr. Vilahur described an experimental animal model in which crossed-bone marrow transplants were performed between Zucker lean and Zucker Fatty/insulin-resistant rats. This study showed that alterations in platelets produced by diabetic bone marrow megakaryocytes contribute to the enhanced thrombotic risk observed in DM [80]. Interestingly, the presence of insulin resistance was already capable of modulating bone marrow released platelets enhancing their susceptibility to form thrombi [81–82]. The work also proved that the bone marrow from diabetic donors induces pro-atherogenic modifications in healthy recipients, increasing their risk of developing atherosclerosis lesions [83].

The low-grade inflammation and oxidative stress seen in DM patients contribute to platelet reactivity through endothelial dysfunction (reduction in eNOS activity) and increased lipid peroxidation to generate F2-isoprostanes which are thought to amplify platelet activation by low concentrations of other agonists. The low-grade inflammatory state triggers IL-6, fibrinogen, and C-reactive protein (CRP) secretion.

Elevated CRP levels constitute an independent risk factor associated with increased cardiovascular mortality in DM [84]. CRP has been shown to enhance the expression of endothelial adhesion molecules, stimulate macrophages to synthesise cytokines, and induce TF expression in monocytes. Additionally, CRP has been reported to modify the fibrinolytic balance of endothelial cells and thus promote fibrin formation, to enhance the expression of PAI-1 in human endothelial cells and inhibit tPA activity. A modified form (monomeric form) of CRP has the ability to contribute to thrombus progression and growth [85–86]. Platelet-derived microparticles (small membrane vesicles released from the surface or plasma membrane of cells upon activation or death) are found at higher levels in people with DM [87] and can exert both pro-inflammatory and pro-thrombotic effects which may contribute to the atherothrombotic response [88–89].

As well as reduced endothelial thrombo-resistance and enhanced platelet activation people with DM often have hypercoagulable blood [90]. Hyperglycaemic states can exert direct effects on the gene transcription of coagulation factors [91] and DM is associated with increased plasma levels and activity of various coagulation factors (TF, factor VII, TF–coagulation factor VIIa complex activity, and factor XII) resulting in enhanced thrombin production. Also, plasma levels of fibrinogen (the soluble precursor of solid fibrin) are increased in diabetes, as part of the ongoing low-grade inflammation. In counterpart, natural anticoagulants such as thrombomodulin, protein C and antithrombin III are found to be reduced in diabetes further developing the prothrombotic environment. Altogether, these changes culminate in increased thrombin generation and fibrin network formation, which is characterised by increased density and improved resistance to fibrinolysis. DM also contributes to thrombotic complications by altering the fibrinolytic mechanisms. As such, diabetic patients have decreased tissue plasminogen activator (t-PA) and enhanced anti-fibrinolytic activity explained through an increase in plasminogen activator inhibitor-1 (PAI-1) and carboxypeptidase B2 (also known as thrombin-activable fibrinolysis inhibitor), thus hampering the conversion of plasminogen to plasmin [92]. Increased levels of PAI-1 are mostly associated with insulin resistance since they are predominantly observed in T2DM patients, but not in other hyperglycaemic situations. Hyperglycaemia can also directly affect the fibrinolytic system by increasing plasminogen glycation (posttranslational modification), thereby adversely affecting its conversion to plasmin [93].

In conclusion, DM is a chronic metabolic disorder in which a hyperglycaemic state and insulin resistance promote a low-grade inflammatory background and systemic oxidative stress which leads to accelerated atherosclerotic progression and an increased risk of thrombosis. Other co-morbidities associated with DM such as obesity, hypertension, and dyslipidaemia further contribute to atherothrombotic complications. The reasons for this adverse cardiovascular profile in people with DM are due to several molecular and cellular pathways that combine to enhance atherosclerosis progression and thrombus formation

**Figure 10.**
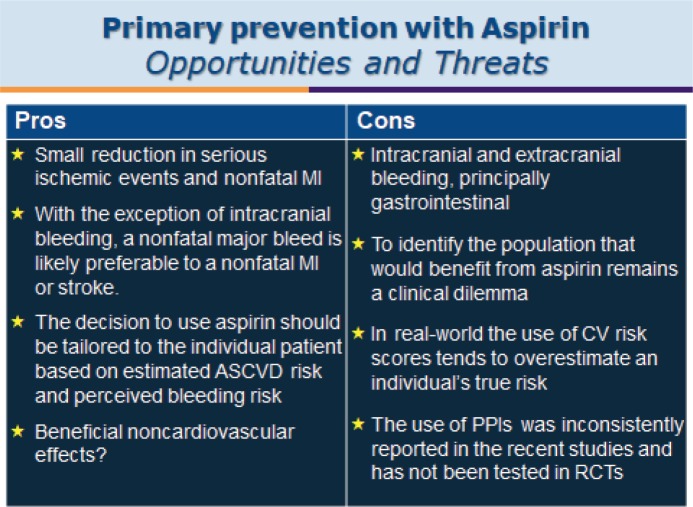
Primary prevention with aspirin: opportunities and threats.

**Prof. Francesco Cosentino (Karolinska Institutet, Stockholm)** gave the final talk of the day providing a very logical journey through primary prevention trials and aspirin in which he looked at the translation of clinical trial evidence into treatment recommendations for aspirin use in diabetes management (Figure 10).

By reducing platelet activity aspirin reduces atherothrombotic events but increases the risk of bleeding events such as GI and intracranial bleeding. In a cohort study of 1.9 million people, 17.9% of people with DMT2 experienced a cardiovascular event over a 5.5-year period [94].

Primary prevention with aspirin is controversial due to the risk-benefit debate. This is of particular concern where the risk of a cardiovascular event is low. There is a high degree of heterogeneity within the trials designed to study the impact of aspirin for primary prevention in general and this has resulted in heterogenicity within the results.

The ASCEND trial [95] showed that, while aspirin did prevent vascular events, it also caused major bleedings. Within ASCEND, one in four of the patients took a PPI and increasing this ratio may reduce bleeding complications.

Prof. Cosentino discussed the issues around whether a cardiovascular event is a worse event than a major bleed. With the exception of intracranial bleeding as long as you do not die of a GI bleed on the whole the experience probably has less impact on a person than having a CVD event. The other complication to using aspirin for primary prevention is that a person’s risk is not static but changes over time; for example, if they adopt a healthier lifestyle their CVD risk reduces. Starting medication to reduce BP or cholesterol will also reduce a person’s overall CVD risk thus impacting the primary prevention risk benefit ratio for aspirin use. Risk is not static.

What has been learnt?

- ‘It is not enough to simply count up cardiovascular events prevented versus bleeding events (most of which are GI ) that have been caused as they do not have the same immediate or eventual health effect.’
- ‘Cost-effectiveness analyses can be helpful provided that we agree with the assumptions used to build the models’
- ‘The greatest potential net benefit occurs in patients with increased cardiovascular risk who are not at increased risk for bleedings (50–59-year-old subjects with a 10-year cardiovascular risk over 10%)’
- ‘Appropriate decision model estimating the ischaemic versus bleeding risk threshold is lacking.’ ‘Perhaps new genetic markers and risk estimators derived from artificial intelligence (AI) may help to refine risk assessment.’

The 2016 European guidelines [96] on CVD prevention in clinical practice with a class 3, level B rating do not recommend antiplatelet therapy for individuals without CVD due to the increased risk of a major bleed.

**Figure 11.**
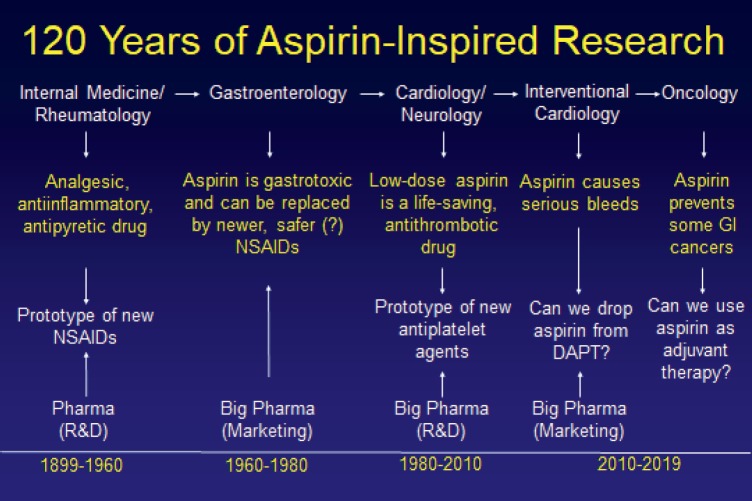
120 years of aspirin- inspired research.

In his concluding remarks to draw this highly interactive and insightful meeting to a close,** Prof. Carlo Patrono (Catholic University, Rome Italy)** summarised 120 years of aspirin inspired research (Figure 11). As newer agents have joined the antithrombotic field some of the research regarding long term disease prevention with aspirin has been passed forward to the oncology community. Aspirin remains a versatile, highly useful drug and it will be interesting to see how its role develops in the future.

## Summary

- In high-risk patients for the secondary prevention of CVD, DAPT with aspirin and a P2Y12 antagonist is more effective in inhibiting platelet aggregation than either drug alone and this is best achieved with the lowest dose of aspirin needed to inhibit more than 95% of platelet TxA2 production; potentially around 30 mg daily.
- Dropping aspirin early on from dual or triple therapy in high-risk patients following a PCI resulted in less bleeding events but more stent thrombosis events.
- In the future, we will have a more targeted approach to CVD management with a better understanding of the variable thrombotic response to atherosclerotic plaque disruption and improved safety/efficacy ratios.
- Clinical judgment is needed to select the patients who have a high CVD risk but a low bleeding risk to benefit most from intensive antithrombotic regimens.
- Aspirin increases the risk of having a GI bleed by both topical and systemic mechanisms and newer antiplatelet drugs carry a similar or slightly higher bleeding risk.
- Adding a PPI or H2RA can reduce the risk of upper GI bleeding. PPIs work better than famotidine (H2RA).
- Aspirin resistance does not exist: it is low compliance and inter-individual variability that affects the response. This variability can be managed with precision dosing.
- A ‘one dose fits all’ approach may not be appropriate for aspirin in the prevention of vascular events or cancer and factors such as body weight and/or BMI may need to be considered.
- Evidence is growing to support the hypothesis that activated platelets contribute to CRC development and metastatic spread. Further research with platelets will be helpful in developing biomarkers for early cancer detection and therapeutic anticancer drug monitoring.
- Long-term follow-up is important in both primary and adjuvant aspirin cancer trials and cancer chemoprevention approaches need wider promotion and greater public awareness.
- DM is a chronic metabolic disorder in which a hyperglycaemic state and insulin resistance promote a low-grade inflammatory background and oxidative stress. This leads to an accelerated atherosclerotic progression and an increased risk of thrombosis.
- The greatest potential net benefit for aspirin primary prevention in CVD exists in those with increased cardiovascular risk who are not at increased risk for bleeding e.g. 50–59-year-old subjects with a cardiovascular risk over 10%.
- A decision-making model that can estimate ischaemic versus bleeding risk is needed.

## Funding declaration

Jaqui Walker was funded by the IAF to attend this event in Rome and received funding for the time required to write the meeting report.

## Conflicts of interest

**Professor Marco Cattaneo**: Research support/P.I: Eli Lilly, Daiichi Sankyo, Astra Zeneca, Evolva. Honoraria: Eli Lilly, Daiichi Sankyo, Astra Zeneca, MSD, Evolva. Scientific Advisory Board: Eli Lilly, Daiichi Sankyo, Astra Zeneca, Evolva, The Medicines Company, MSD.

**Professor Lina Badimon**: Consultant and speaker fees from Sanofi, AstraZeneca, Amgem. Grant support for investigator-initiated research from: European Commission, FP6 and FP7 programmes, National Funding Agencies, AstraZeneca.

**Professor Giancarlo Agnelli**: Speaker bureaux: Bristol-Myers Squibb/Pfizer, Bayer Healthcare. Advisory boards: Bristol-Myers Squibb/Pfizer, Daiichi Sankyo, Bayer Healthcare.

**Professor Andrew Chan**: Consultant for Pfizer Inc, Bayer Pharma AG, Janssen. Grants for investigator-initiated research National Institute of Health, National Cancer Institute, Crohn’s and Colitis Foundation, Bayer AG. 2019 American Association for Cancer Research AACR-Waun Ki Hong Award.

**Professor Angel Lanas**: none.

**Professor Bianca Rocca**: Institutional research grants, research grants from the Italian Medicines Agency (AIFA), consultancy from Bayer AG.

**Professor Peter Rothwell**: Consultant and speaker fees from Bayer AG.

**Professor Paola Patrignani**: Grant Associazione Italiana per la Ricerca sul Cancro (AIRC, IG-12111), Aspirin for Cancer Prevention Group (AsCaP), Bayer for the PK/PD studies with EC-ASA, Fellowship to Dr Angela Sacco by SIF-Merck 2017.

**Professor Ruth Langley**: Honorarium Bayer, clinical trial drug supplies for an academic study.

**Dr Gemma Vilahur**: Speaker fees from AstraZeneca, research grants from National Funding Agencies.

**Professor Francesco Consentino**: Research grants: Swedish Research Council, Swedish Heart and Lung foundation, Karolinska Institutet, European Foundation for the Study of Diabetes, Swedish Diabetes Foundation, King Gustav V and Queen Victoria Foundation. Advisory board/speaker: AstraZeneca, Boehringer Ingelheim, Bristol Myers Squibb, Eli Lilly, Merck Sharp and Dohme, Mundipharma, Novo Nordisk, Pfizer.
